# Dissemination of scientific software with Galaxy ToolShed

**DOI:** 10.1186/gb4161

**Published:** 2014-02-20

**Authors:** Daniel Blankenberg, Gregory Von Kuster, Emil Bouvier, Dannon Baker, Enis Afgan, Nicholas Stoler, James Taylor, Anton Nekrutenko

**Affiliations:** 1 Department of Biochemistry and Molecular Biology, Penn State University, University Park, PA 16802, USA; 2 Departments of Biology and Computer Sciences, Johns Hopkins University, Baltimore, MD 21218, USA; 3 Interdisciplinary Graduate Program in BioSciences, Penn State University, University Park, PA 16802, USA; 4 Galaxyproject.org, University Park, PA 16802, USA and Baltimore, MD 21218, USA; 5 Center for Informatics and Computing, Ruđer Bošković Institute, Zagreb 1000, Croatia

## Abstract

The proliferation of web-based integrative analysis frameworks has enabled users to perform complex analyses directly through the web. Unfortunately, it also revoked the freedom to easily select the most appropriate tools. To address this, we have developed Galaxy ToolShed.

## 

Previously, our group has investigated the persistence of mitochondrial variants (heteroplasmies) through mother-child transmissions [1]. Many disease-causing mitochondrial variants are heteroplasmic and their clinical manifestations depend on the relative proportion of normal to mutant alleles [2-4]. Because almost all of the mitochondrial genome is transcribed [5], the next important question is whether the relative frequencies of heteroplasmic alleles are maintained in transcripts. We turned to published studies to find the appropriate dataset that would include matched genomic and transcriptomic data. The initial analysis of DNA/RNA differences by Li *et al*. [6] omitted the mitochondrial transcriptome and a much more comprehensive dataset by Chen *et al*. [7] has since become available. The latter contains both whole genome and RNA sequencing data from a single individual and is therefore ideally suited for our purpose. To perform this analysis, we started with a ‘clean’ Galaxy Amazon EC2 instance [8-10], mapped the reads against the latest version of the human genome, retained properly mapped pairs, removed reads mapping to multiple locations, added readgroup information, and combined all results into a single binary version of the sequence alignment/map format (BAM) dataset for further analysis (Additional file 1) [11].

At this point in the analysis, we ran into the first roadblock: the Galaxy instance we were using did not contain any tools for detecting sequence variants. This is exactly the type of situation where the ToolShed is the most useful, as it already contains a collection of utilities for variant detection such as FreeBayes [12]. Installing the FreeBayes tool along with the required dependencies into Galaxy using the ToolShed is accomplished through the web-based graphical user interface [11]. Behind the scenes, the ToolShed fetches source code from the FreeBayes GitHub repository, compiles it, and registers all necessary components with the Galaxy instance, making it accessible to the user [13]. Application of FreeBayes to our dataset has identified two potential heteroplasmic sites with minor allele frequencies >2% (a heteroplasmy detection threshold derived from empirical and simulation data [1,14]): 2,619 and 13,636 (Figure 1a,b). Site 13,363 is a textbook example of a heteroplasmy - it is biallelic (T/C) with an average minor allele frequency of 22% across the 21 samples in our study. However, the other site, 2,619, is different and represents a potential RNA modification reported recently by our group [15]. Within genomic DNA it is represented by an invariable A, while in all RNA-seq datasets it is scored by FreeBayes as a heterozygous locus with the major allele being a T. Moreover, while the total coverage at this site across all samples was 40,132, the numbers of reference and alternative observations were 11,086 and 20,584, respectively (summing to a total of 31,670), suggesting that the site is multiallelic. FreeBayes used here only reports two possibilities: reference and alternative. However, in many cases, such as genotyping of pooled, bacterial or viral samples, it is necessary to report exact counts for all variants. In a typical sequence analysis experiment this is the point where custom scripts are often being developed. While we did exactly that - developed two custom Python-based tools, ‘Naïve Variant Caller’ (NVC) and ‘Variant Annotator’ - we went a step further and deposited these tools into the ToolShed. By doing so, we not only made it accessible to any Galaxy instance, but also ensured reproducibility of our experiment, which is almost universally lacking in studies utilizing custom scripts [16]. The NVC produces Variant Call Format (VCF) output [17] containing counts for all observed variants from multisample BAM datasets (Additional file 1), while Variant Annotator converts VCF data into allele counts stratified by samples. To deposit the tools into the ToolShed, we have created a version-controlled repository and uploaded all software components, including the tool configuration file, NVC Python script, information about necessary software dependencies, and a set of functional tests. At this point, the tool becomes ‘visible’ to any Galaxy installation, including the cloud-based instance we use in this study. After installing the NVC from the ToolShed [18], we have applied it to the original BAM dataset to obtain counts shown in Figure 1c,d. Here the multiallelic nature of site 2,619 is clearly seen as well as the fact that this variation only appears in transcriptome data.

**Figure 1:**
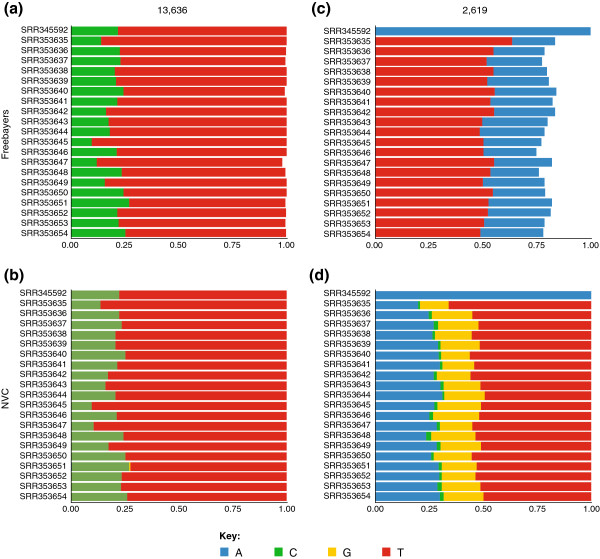
**Frequency of the four possible nucleotides across genomic DNA (accession number SRR345592) and RNA-seq (accession numbers SRR353635-SRR353654) samples for sites 13,636 and 2,619.** NVC, Naïve Variant Caller. Data is deposited in the Short Read Archive at the National Center for Biotechnology Information (NCBI).

This short example has illustrated that the ToolShed behaves as a *de facto* AppStore: when users need an analysis tool that is not present in a given Galaxy instance, it can be easily fetched and installed. Just like a brand new iPad, Galaxy comes with a small number of preinstalled applications providing basic functionality. Additional tools may subsequently be installed from the ToolShed to create a ‘flavor’ of Galaxy suitable for a particular analysis. An expanded discussion of the ToolShed can be found in the online supplement.

### Abbreviations

BAM: Binary version of the sequence alignment/map format; NVC: Naïve Variant Caller; VCF: Variant call format.

### Competing interests

The authors declare that they have no competing interests.

## Supplementary Material

Additional file 1 Contains examples of tools deposited to ToolShed and discusses implications of this system for improving the reproducibility of biomedical research. Click here for file
