# Supplementary material for: Dissemination of scientific software with Galaxy ToolShed
Source: Genome Biol. 2014 Feb 20;15(2):403. doi: 10.1186/gb4161 (PMC4038738; doi:10.1186/gb4161)
Supplement: Additional file 1 — Contains examples of tools deposited to ToolShed and discusses implications of this system for improving the reproducibility of biomedical research. [file gb4161-S1.pdf]

Tool Complexity

The tools highlighted in the main text illustrate various level of tool complexity. The Variant Annotator (http://goo.gl/SLJwkF) is a stand-alone Python script with no dependencies that can easily be installed into Galaxy running on almost any platform. The `NVC` (http://goo.gl/QVQcp9) is also a Python-based tool. However, it requires several Python packages including the scientific computing library NumPy [1] and several modules for manipulation of BAM files. The ToolShed configuration file for `NVC` contains instructions necessary for installation of these dependencies. This enables Galaxy, for instance, to download the NumPy package from its native URL and to compile it for a platform running any given Galaxy installation (such as the one on the AWS utilizing the Ubuntu operating system). FreeBayes, on the other hand, is a very different kind of analysis utility (http://goo.gl/26yuLj). To install FreeBayes, its C++ source code needs to be downloaded from GitHub and built with g++ compiler while noting that some of its components require the cmake utility. In addition, a particular version of SamTools [2] needs to be built alongside FreeBayes, and appropriate environmental variables have to be configured for the newly installed tool to be accessible for a Galaxy instance. The ToolShed contains configuration syntax that makes this possible. While the three tools provide an overview of the ToolShed's configuration syntax, they do not illustrate the full extent of possible complexities. One of the largest and most advanced sets of tools available in the ToolShed is represented by the ChemicalToolBoX (http://goo.gl/lAfxLv; Gruning et al. Submitted). The ChemicalToolBoX is a collection of 32 tools depending on over 20 external packages. Another intriguing feature of the ChemicalToolBoX is that it is not a genomic set of utilities, but a collection of computational chemistry tools. Other examples of complex tool suites available from the ToolShed include the metagenomic packages mothur [3] and QIIME [4], each containing close to a hundred individual tools and integrated by community contributors.

Reproducibility and tool versioning

Low overall reproducibility of published results represents a significant challenge for today's biomedical research, effectively blocking scientific progress [5, 6]. In fact, reproducibility is an ensemble of related, but independent, issues ranging from providing access to primary data to recording exact details of every analytical procedure. One of the most challenging aspects of making biomedical analyses repeatable is managing versions of the tools used to interpret data. This is because software evolves continuously and the latest versions of any given tool may not produce the same results as an earlier one. Fig. S1 shows variation in allele frequency at a human mitochondrial site depending upon which version and parameter combination of a widely used short read mapper, bwa [7], has been used in the analysis. One can see that the earlier versions have been particularly problematic. In this regard, making every Galaxy analysis reproducible meant keeping every version of every tool in all existing instances throughout the world, which is not practical. The ToolShed solves this challenge by providing a centralized tool versioning system. Because every Galaxy tool is versioned, repeating analyses becomes possible even when a particular instance is

missing the correct tool: the user is warned that the current version of a tool is different and is provided with an option of installing the correct version from the ToolShed.

Ensuring quality of ToolShed submissions

Anyone can submit to the ToolShed, which now contains over 2,000 tools. The idea behind such openness is decreasing the initial barriers for faster adoption by the community. This approach has worked well for us before, yet it has one significant disadvantage. With submission being straightforward (http://goo.gl/1cKagk), there is no reward for quality, making it difficult for end users to differentiate between good tools and low quality submissions. To deal with this situation, we have established the Intergalactic Utilities Commission (IUC), consisting of Galaxy tool developers from US, Europe, and Australia, tasked with reviewing and flagging high quality tools. To simplify the work of the IUC, we have developed a suite of ToolShed components designed to automatically evaluate submissions prior to formal IUC review. These components include a series of scripts that verify the existence of test data, and execute the functional tests defined within the tool configuration.

Beyond Tools

As more and more local and cloud-based Galaxy instances are being used, there is a need for a central hub that would serve as a middle ground for storing and exchanging analytical components. We view the ToolShed as such a hub. In this report, we described its functionality in regard to handling analysis tools. However, it already extends beyond tools to include workflows. When one installs a workflow, the ToolShed automatically installs tools that are needed for the workflow but are missing from a given Galaxy instance. In the future, we will extend the ToolShed to provide a centralized repository of tools, data, metadata, analysis workflows and practices as well as their published descriptions in the form of Galaxy Pages, which will be linked to relevant journal articles, providing an unprecedented level of research reproducibility and transparency.

Methods

Accessing data and tools

A new Galaxy instance on Amazon can be instantiated by pointing a web browser to http://usegalaxy.org, selecting “Cloud” on the upper pane of the interface, and clicking on the “New Cloud Cluster” link (the prodedure is also detailed at http://usegalaxy.org/cloud). The following screencasts detail all ToolShed aspects described in this manuscript:

1. Screencast 1 (https://vimeo.com/73458993) explains how to start a new instance of Galaxy on Amazon’s EC2 cloud and populate it with data

2. Screencast 2 (https://vimeo.com/73460697) covers installation of the FreeBayes tool from the ToolShed and generation of raw variant calls.

3. Screencast 3 (https://vimeo.com/73462389) highlights the installation of the Naive Variant Caller and Variant Annotator tools and their application to the Chen et al. [8] data.

In addition, we provide a BAM file containing reads from blood (SRR345592) and twenty RNA-seq time-points (SRR353635 - SRR353654) aligned against the hg19 version of the human genome (http://goo.gl/puWbOC). This file was prepared in the following way. First, reads from individual samples were aligned against the hg19 version of the human genome within Galaxy using bwa version 0.5.9-r16. The following settings were used for the bwa aln command: -n 0.04 -o 1 -e -1 -d 16 -i 5 -l -1 -k 2 -M 3 -O 11-E 4. Subsequently, the bwa sampe command was executed using these flags: -n 3, -N 10, -a 500, -o 100000 and specifying readgroups for each sample. Individual SAM files were merged into a single BAM file in Galaxy using the Picard (http://picard.sourceforge.net/) mergeSam command and filtered to remove inconsistent read pairs and to restrict final datasets to only those reads that are mapped to the mitochondrial genome (chrM).

Naive Variant Caller

The Naive Variant Caller (http://goo.gl/QVQcp9) processes aligned sequencing reads from the BAM format and produces a VCF file containing per position variant calls. This tool allows multiple BAM files to be provided as input and utilizes read group information to make calls for individual samples. User configurable options allow filtering reads that do not pass mapping or base quality thresholds and minimum per base read depth; users can also specify the ploidy and whether to consider each strand separately. In addition to calling alternate alleles based upon simple ratios of nucleotides at a position, per base nucleotide counts are also provided. A custom tag, NC, is used within the Genotype fields. The NC field is a comma-separated listing of nucleotide counts in the form of <nucleotide>=<count>, where a plus or minus character is prepended to indicate strand, if the strandedness option was specified.

Variant Annotator

The Variant Annotator (http://goo.gl/SLJwkF) processes the raw variant count data from the Naive Variant Caller. Single nucleotide variant counts and allele statistics are reported for each site in a simple tabular format. Data from multiple samples are supported, via sample columns in the input VCF. The first and second most abundant variants are reported, along with the frequency of the latter. The user can set a coverage threshold, which is applied to each strand individually. An allele count is computed, based on the number of alleles passing a user-supplied frequency threshold. A basic filter for strand bias is applied at this stage, excluding sites where the threshold-passing alleles differ between the strands. At these sites, neither allele count is used, and the tool will instead mark it zero.

Figure S1. Observed frequency fluctuation (Y-axis) at site 8,992 of human mitochondrial genome [9] as a function of bwa version (X-axis) and parameter settings.

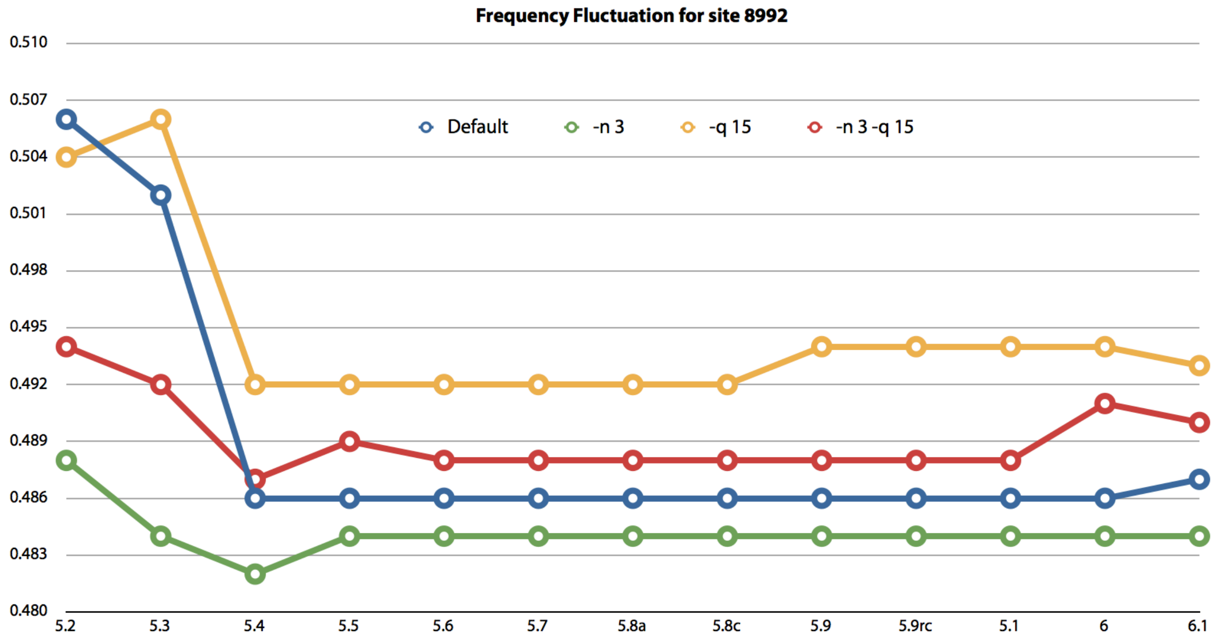

References

1. van der Walt S, Colbert SC, Varoquaux G: **The NumPy Array: A Structure for Efficient Numerical Computation**. *Comput Sci Eng* 2011, **13**:22–30.

2. Li H, Handsaker B, Wysoker A, Fennell T, Ruan J, Homer N, Marth G, Abecasis G, Durbin R, 1000 Genome Project Data Processing Subgroup: **The Sequence Alignment/Map format and SAMtools.** *Bioinformatics* 2009, **25**:2078–2079.

3. Schloss PD, Westcott SL, Ryabin T, Hall JR, Hartmann M, Hollister EB, Lesniewski RA, Oakley BB, Parks DH, Robinson CJ, Sahl JW, Stres B, Thallinger GG, Van Horn DJ, Weber CF: **Introducing mothur: Open-Source, Platform-Independent, Community-Supported Software for Describing and Comparing Microbial Communities**. *Appl Environ Microbiol* 2009, **75**:7537–7541.

4. Caporaso JG, Kuczynski J, Stombaugh J, Bittinger K, Bushman FD, Costello EK, Fierer N, Peña AG, Goodrich JK, Gordon JI, Huttley GA, Kelley ST, Knights D, Koenig JE, Ley RE, Lozupone CA, McDonald

D, Muegge BD, Pirrung M, Reeder J, Sevinsky JR, Turnbaugh PJ, Walters WA, Widmann J, Yatsunenko T, Zaneveld J, Knight R: **QIIME allows analysis of high-throughput community sequencing data**. *Nat Methods* 2010, **7**:335–336.

5. Ioannidis JPA, Allison DB, Ball CA, Coulibaly I, Cui X, Culhane AC, Falchi M, Furlanello C, Game L, Jurman G, Mangion J, Mehta T, Nitzberg M, Page GP, Petretto E, van Noort V: **Repeatability of published microarray gene expression analyses**. *Nat Genet* 2009, **41**:149–155.

6. Nekrutenko A, Taylor J: **Next-generation sequencing data interpretation: enhancing reproducibility and accessibility**. *Nat Rev Genet* 2012, **13**:667–672.

7. Li H, Durbin R: **Fast and Accurate Short Read Alignment with Burrows-Wheeler Transform**. *Bioinformatics* 2009:1–7.

8. Chen R, Mias GI, Li-Pook-Than J, Jiang L, Lam HYK, Chen R, Miriami E, Karczewski KJ, Hariharan M, Dewey FE, Cheng Y, Clark MJ, Im H, Habegger L, Balasubramanian S, O'Huallachain M, Dudley JT, Hillenmeyer S, Haraksingh R, Sharon D, Euskirchen G, Lacroute P, Bettinger K, Boyle AP, Kasowski M, Grubert F, Seki S, Garcia M, Whirl-Carrillo M, Gallardo M, et al.: **Personal Omics Profiling Reveals Dynamic Molecular and Medical Phenotypes**. *Cell* 2012, **148**:1293–1307.

9. Goto H, Dickins B, Afgan E, Paul IM, Taylor J, Makova KD, Nekrutenko A: **Dynamics of mitochondrial heteroplasmy in three families investigated via a repeatable re-sequencing study.** *Genome Biol* 2011, **12**:R59.
